# Maternal protein deficiency alters primary cilia length in renal tubular and impairs kidney development in fetal rat

**DOI:** 10.3389/fnut.2023.1156029

**Published:** 2023-07-06

**Authors:** Jun Wang, Pei Zhou, Liangliang Zhu, Hongbo Guan, Jian Gou, Xiaomei Liu

**Affiliations:** ^1^Department of Obstetrics and Gynecology, Shengjing Hospital of China Medical University, Shenyang, China; ^2^Department of Nutrition, Shengjing Hospital of China Medical University, Shenyang, China

**Keywords:** FGR, kidney, primary cilia, IFT88, DYNLT1, β-catenin pathway

## Abstract

**Introduction:**

Intrauterine malnutrition impairs embryo kidney development and leads to kidney disease and hypertension in adulthood, yet the underlying mechanism remains unclear.

**Methods:**

With a maternal protein restriction (MPR) rat model, we investigated the critical ciliogenesis factors and β-catenin pathway in FGR fetal kidneys and analyzed the impact of aberrant primary cilia on renal tubular epithelium.

**Results:**

The data showed decreased nephron number and renal tubular dysgenesis in FGR fetus. FGR fetus showed deregulated expression of ciliogenesis factors including upregulation of IFT88 and downregulation of DYNLT1, accompanied with cilia elongation in renal tubular epithelial cells. Wnt7b, the key ligand for Wnt/β-catenin signaling, was downregulated and nuclear translocation of β-catenin was decreased. The proapoptotic protein was upregulated. *In vitro* study with HK-2 cells showed that overexpression of IFT88 lengthened the cilia, inhibited β-catenin signaling. Besides, IFT88 overexpression suppressed cell proliferation, activated autophagy, and induced cell apoptosis. Inhibition of autophagy partly restored the cilia length and cell viability. Likewise, knockdown of DYNLT1 led to cilia elongation, suppressed cell proliferation, and promoted apoptosis in HK-2 cell. However, the cilia elongation induced by DYNLT1 knockdown was not autophagy-dependent, but associated with reactive oxygen species (ROS) accumulation.

**Discussion:**

We elucidated that intrauterine protein malnutrition led to deregulation of ciliogenesis factors and cilia elongation in renal tubular epithelial, inhibited β-catenin signaling, and induced cell apoptosis and ultimately, compromised kidney development.

## 1. Introduction

Fetal growth restriction (FGR) is defined as a failure of a fetus to reach its intrauterine potential for growth and development, which affecting around 5–10% of pregnancies (1, 2). FGR increases risks for preterm birth and neonatal morbidity, and also adulthood diseases such as hypertension, diabetes and cardiovascular disease (3). Epidemiological investigations have revealed that chronic kidney disease is closely associated with low birth weight (4). Studies in animal models confirm that FGR leads to decreased nephrons, reduced glomerular filtration rate, and aberrant sodium ion excretion, which are closely related to the occurrence of kidney disease and hypertension in adulthood (5, 6). Nevertheless, pathophysiological mechanisms underlying these changes are still largely unknown.

Primary cilia are small, membrane-enclosed organelles that ubiquitously present in mammalian cells. Many signaling pathway proteins, such as polycystins (alias polycystic kidney disease, PKD), Wnt pathway[β-catenin and inversin], and Shh pathway are localized to primary cilia (7, 8). It orchestrates signal transduction in various cells, by sensing signals, such as hormones and fluid flow, in the microenvironment and transmitting the signals to the cytoplasm. Defects in ciliogenesis and function result in “ciliopathies,” usually manifested with abnormal kidney architecture or function (8). Primary cilia regulate canonical (β-catenin-dependent) and non-canonical (β-catenin-independent) Wnt signaling, thus regulate cell proliferation and death. Wnt/β-catenin signaling plays a key role in controlling early nephrogenesis, and is implicated in the pathogenesis of various kidney disorders (9, 10). β-catenin signaling is crucial for ureteric bud (UB) induction, nephron formation and maturation. Wnt7b regulates neovascularization around UBs of the medulla, and Wnt9b induces the differentiation of UB cells (11, 12). The assembly and maintenance of cilia depend on intraflagellar transport (IFT) proteins (13). We previously revealed that maternal protein deficiency led to upregulation of the ciliogenesis factors IFT88 and PKD in FGR offspring (14). Downregulation of IFT88 was reported to activate Wnt pathway and promote β-catenin nuclear translocation (15). Cilia dysfunction caused by Pkd1 deletion also causes renal Wnt7a/7b overexpression and activates Wnt/β-catenin pathway (16). To date, there are few reports on ciliogenesis or Wnt/β-catenin pathways in FGR, the only relevant study reported that maternal dexamethasone exposure leads to a reduction in β-catenin activity and inhibition of Wnt signaling in the placenta (17). Whether cilia-dependent β-catenin pathway is involved in renal dysplasia in FGR fetus is unknown.

In the current investigation, we tested the hypothesis that deregulation of ciliogenesis factors affected the morphology and function of primary cilia, subsequently inhibited canonical Wnt/β-catenin signaling, thereby impaired embryo kidney development in FGR rats. The present study investigated the changes of ciliogenesis factors, β-catenin signaling pathway and apoptosis regulators in fetal kidneys of FGR rats. To further investigate this hypothesis, we used HK-2 cell to study the impacts of IFT88 overexpression and DYNLT1 deficiency on β-catenin signaling and cell viability in human proximal tubular epithelial cell.

## 2. Materials and methods

### 2.1. Animal model

The FGR model was established as previously described (14). In brief, Wistar rats (Changsheng Biotechnology, Liaoning, China) weighing 230–260 g were housed under specific pathogen-free conditions with constant temperature and relative humidity. Time-pregnant rats were randomly divided into two groups: animals in maternal protein restriction (MPR) group were fed an isotonic low-protein diet throughout pregnancy, and animals in the control (CON) group were fed a normal diet (Supplementary Table S1). A set of pups was delivered by caesarean section on day 20 of gestation (E20). A fetus with a body weight of two standard deviations less than the mean body weight of the control group was classified as FGR, according to the definition of human FGR. The offspring rats whose birth weight did not meet the FGR criteria were excluded, and the remaining offspring were fed under the normal diet until 12 weeks (12 W). At 3 W and 12 W, offspring rats of both groups were killed under ether anesthesia and the kidneys were collected. The right kidney was fixed in 4% paraformaldehyde and left kidney was stored at −80°C until analysis. At 12 W, a non-invasive rat-tail pressure gauge was used to record the systolic blood pressure, and a metabolic cage was used to obtain 12 h urine for renal functional parameter tests (Abbott, ARCHITECT ci16200, Chicago, United States).

### 2.2. Quantitative PCR

Total RNA was extracted with Trizol (Invitrogen) according to the manufacturer protocol, and reverse transcribed into cDNA as described previously (14). Q-PCRs were performed using a SYBR Green PCR Kit (Vazyme, Q711-02, Nanjing, China) with the primers described in Supplementary Table S2. Experiments were carried out in triplicate. β-actin was used as the housekeeping gene. Relative expression level was determined as 2^−ΔΔCT^. ΔCT was calculated as ΔCT = Avg Target CT − Avg Housekeeping CT. 2^−(ΔΔCt)^ = 2^−(Experimental ΔCt − Control ΔCt)^.

### 2.3. Western blot

Total protein was extracted using SDT lysis buffe and measured using a BCA assay kit (Pierce Biotechnology, Waltham, MA, United States). Equal amounts of proteins were separated by 6–12% polyacrylamide gel electrophoresis and transferred onto a polyvinylidene difluoride membrane. The membrane was cut into several strips, blocked with 5% bovine serum albumin, and probed with indicated primary antibodies at 4°C overnight (Supplementary Table S3). After incubated with horseradish peroxidase-conjugated secondary antibodies, the antigen–antibody complexes were visualized using enhanced chemiluminescence plus reagent (Millipore, Billerica, MA, United States). Experiments were carried out in triplicate. Gel-Pro Analyzer (Bio-Rad) was used for band densitometry, using β-actin as an internal reference. Relative quantification of protein levels was expressed as fold change relative to the control.

### 2.4. Histology and immunostaining

Kidneys collected at three time points (E20, 3 W and 12 W) were fixed with 4% paraformaldehyde, dehydrated, and paraffin-embedded. 3 μm-sections were used for periodic acid–Schiff (PAS) staining (Solarbio, Beijing, China), immunohistochemistry (IHC) and immunofluorescence (IF) staining, according to standard procedures with primary antibodies (Supplementary Table S1). For PAS staining, the numbers of glomeruli in at least 5 fields per kidney section were counted to quantify the change of nephron between the two groups. For IHC and IF staining, sections, or cell coverslips were antigen-repaired, blocked of endogenous peroxidase (3% hydrogen peroxide, 30 min) and non-specific antigen (5% serum, 30 min), and incubated with primary antibody at 4°C overnight. A negative control without the primary antibody was included, and no unwanted background staining was observed. Sections, or cell coverslips were incubated with biotin-conjugated secondary antibody and then developed with DAB (for IHC), or incubated with fluorescent-conjugated secondary antibody in the dark, followed by nuclear staining with 4′,6-diamidino-2-phenylindole (DPAI, for IF). Images of IHC were acquired using a Nikon ECLIPSE Ti microscope, and images of IF were acquired with a Nikon C1 confocal microscope. The mean fluorescence intensity (MFI) was analyzed by ImageJ software and calculated from three slides per group. For each slide, three representative sections were scored.

### 2.5. Cell culture and transfections

HK-2 cells (a human proximal tubular epithelial cell line) were cultured at 37°C in 5% CO_2_ atmosphere in DMEM-F12 medium (Procell, Wuhan, China) supplemented with 10% fetal bovine serum (Procell) and 1% penicillin/streptomycin solution. Cells were plated according to the requirement of each experiment as described below. Plasmid overexpression of IFT88 (pGV417-IFT88) was purchased from GeneChem (Shanghai, China). The plasmid was transformed into the *E. coli* DH5α and purified using EndoFree Maxi Plasmid Kit (Tiangen, Beijing), according to the manufacturer’s manual. The presence of plasmid DNA was confirmed by DNA sequencing. SiRNAs targeted for DYNLT1 and Control siRNA (si-NC) were purchased from Sangon (Shanghai, China). The sequences of siRNA and PCR identification primers for pGV417-IFT88 were listed in Supplementary Table S2. For oligonucleotides transfection, cells were transfected at 70–80% confluence (24 h after plating) using siRNA or scramble control(si-NC) and INTERFERin (Polyplus Transfection), according to the manufacturers’ protocol. The IFT88 overexpression plasmid (pGV417-IFT88) as well as the corresponding empty plasmid (p-MOCK) were transfected using Lipofectamine 3,000(L3000015, Thermo). Silence or overexpression was confirmed on the RNA and protein levels. For apoptosis activation or inhibition assay, the cells were treated separately with 200 nM staurosporine (STS, apoptosis activator) or 20 μM Z-DEVD-FMK (caspase 3 inhibitor). For autophagy inhibition experiments, the cells were treated with 5 mM 3MA (autophagy inhibitor).

### 2.6. Cell proliferation assay

EdU staining was conducted with an EdU Kit (Beyotime, Shanghai, China), following the manufacturer’s instruction. Briefly, cells were incubated with 10 μM EdU for 2 h, fixed with paraformaldehyde and then stained with anti-EdU Alexa Fluor 488 and DAPI. Images were captured with an inverted fluorescence microscope (Nikon, Tokyo, Japan). Double-stained cells were considered EdU-positive (EdU+) cells, which indicate cells at S phase. EdU-labeled cells (green fluorescently labeled cells) and non-labeled cells were counted, and the percentage of EdU+ cells was calculated.

For CCK8 assay, cells were plated in 96-well plates at a density of 5 × 10^3^ cells/well, and then added with CCK8 solution and incubated for 2 h at 37°C. Then the absorbance was immediately measured at 450 nm using a microplate reader, while cell viability was calculated accordingly.

### 2.7. Apoptosis assay

In siRNA transfection cells, apoptotic rate was determined using Annexin V-FITC/PI kit (Elabscience, Wuhan, China), according to the manufacturer’s instructions. Apoptosis in HK2 cells was induced by 400 nmol/L staurosporine (A8192, APExBIO, United States) for 24 h. Cells were washed with cold PBS, resuspended in binding buffer, and then mixed with 5 μL of Annexin V-FITC and 5 μL of PI. The cells were incubated for 15 min at room temperature in the dark and then analyzed using a FACScan flow cytometer (BD Bioscience). Apoptosis is defined as those cells that positively staining of Annexin with or without PI, and the data are expressed as the percentage of these cells per total cells counted.

In plasmid transfection cells, apoptotic rate was determined using TUNEL staining (Beyotime, Shanghai, China), not Annexin V-FITC/PI staining, for the p-Mock plasmid expressing the red fluorescent protein mCherry. Briefly, cells were fixed with 4% paraformaldehyde, permeabilized with Triton X-100, and then subjected to the TUNEL assay. Nuclei were stained with DAPI. Images were acquired with a laser-scanning confocal microscope. The cells with green fluorescence were considered as apoptotic cells. TUNEL-labeled cells (green fluorescently labeled cells) and non-labeled cells were counted, and the percentage of TUNEL+ cells was calculated.

Caspase 3/7 activity was assayed with GreenNuc™ Caspase-3 Assay Kit (Beyotime). Cells were incubated with 5 μM GreenNuc™ Caspase-3 substrate in culture medium for 30 min at 37°C in the dark, then counterstained with Hoechst33342. Fluorescence images were obtained with a confocal microscope.

### 2.8. Detection of autophagic flux

Autophagy flux was determined with the Cyto-ID^®^ Autophagy detection kit (Enzo, ref. ENZ-51031), according to the manufacturer’s protocols. HK-2 cells were grown on coverslips and transfected with p-IFT88 or si-DYNLT1. After 48 h cells were incubated with the CYTO-ID green reagent for 30 min at 37°C in the dark, then images were acquired with a confocal microscopy. Mean fluorescence intensity was quantified using Image J software.

### 2.9. Intracellular reactive oxygen species detection

Cytosolic ROS level was measured with the fluorescent probes 5-(and-6)-chloromethyl-2-,7-dichlorodihydrofluorescein diacetate (DCHF-DA). Cells were collected and incubated with DCHF-DA (10 μM, 30 min) at 37°C in the dark according to the manufacturer’s protocol. Cells treated with H_2_O_2_ (100 mM) was used as a positive control. For fluorescence quantitative analysis, the cells were collected and analyzed by flow cytometry.

### 2.10. Statistical analysis

Statistical analysis was performed using GraphPad Prism 5 software (Graphad Software Inc., San Diego, United States). Data are presented as the mean ± standard error of the mean (SEM). All data were checked for normal distribution and homogeneity of variance using the Shapiro–Wilk test and the Levene test, respectively. Two-tailed Student’s unpaired *t*-test was used for comparison between two groups, while one-way ANOVAs was used for multiple groups comparison. Differences in mean values were considered significant at *p* < 0.05.

## 3. Results

### 3.1. Effects of MPR on kidney function index and histopathology

**Figure 1:**
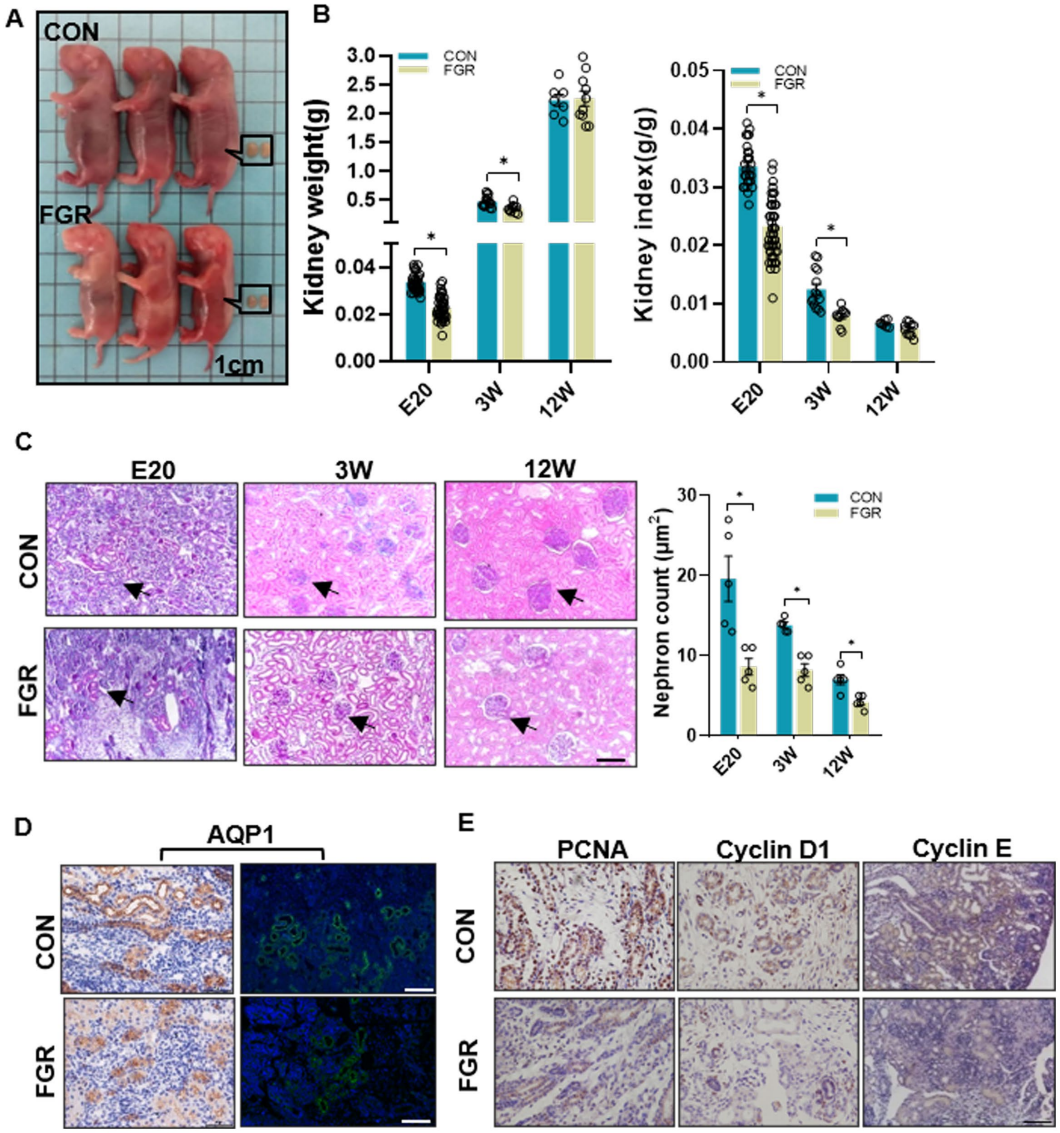
Effect of maternal protein restriction on offspring kidney development. **(A)** Representative photos of fetuses and fetal kidneys of FGR and control groups. **(B)** Kidney weight and kidney index at the indicated time points in both groups. (E20, FGR *n* = 52, CON *n* = 30; 3 W, FGR *n* = 10, CON *n* = 14; 12 W, FGR *n* = 8, CON *n* = 7; *p < 0.05 vs. same-aged control). **(C)** PAS-staining showing a decrease in nephron numbers. Black arrow indicate nephron. Images were taken at a magnification of 200×; scale bar = 50 μm (*n* = 5, *p < 0.05 vs. control). **(D)** IF and IHC staining of AQP1 in fetal kidney (400×, scale bar =100 μm). **(E)** IHC staining of the proliferation marker PCNA and cell cycle regulators Cyclin D1 and Cyclin E (400×, scale bar = 100 μm).

We previously reported that in FGR group, the body weight was reduced in fetus (E20), restored to normal level after weaning (3 W) due to catch-up growth, and surpassed that of the same aged-control in adulthood (12 W) (18). Compared to control, the kidney weight of FGR offspring was decreased from E20 to 3 W, restored to normal at 12 W, whereas the renal index remained lower than control at 12 W (Figures 1A,B). At 12 W, systolic blood pressure was significantly higher in FGR group (Supplementary Table S4). Urinary biomarkers, N-acetyl-β-D-glucosaminidase, urine protein, and blood urea were slightly increased, which indicated the presence of renal dysfunction (Supplementary Table S4). PAS staining showed that the number of renal cortical glomeruli was significantly lower in the FGR fetus than in the control group, and the dysplasia phenotype lasted until adulthood (12 W) (Figure 1C). Aquaporin (AQP)1, a proximal tubular epithelium marker, was decreased in FGR fetal kidney (Figure 1D), suggestive of renal tubular dysgenesis. PCNA, a well-accepted cell proliferation marker was downregulated in the kidney of FGR group, suggestive of decreased cell proliferation. Cyclin D1 and Cyclin E, two positive regulators of the transition from G1 to S phase, were also reduced (Figure 1E), which may cause abnormal cell cycle progression and lead to decreased cell proliferation. The above results indicated the dysplasia of the renal tubular and glomerular in FGR fetus.

### 3.2. Effects of MPR on primary cilia regulators in fetal kidney

**Figure 2:**
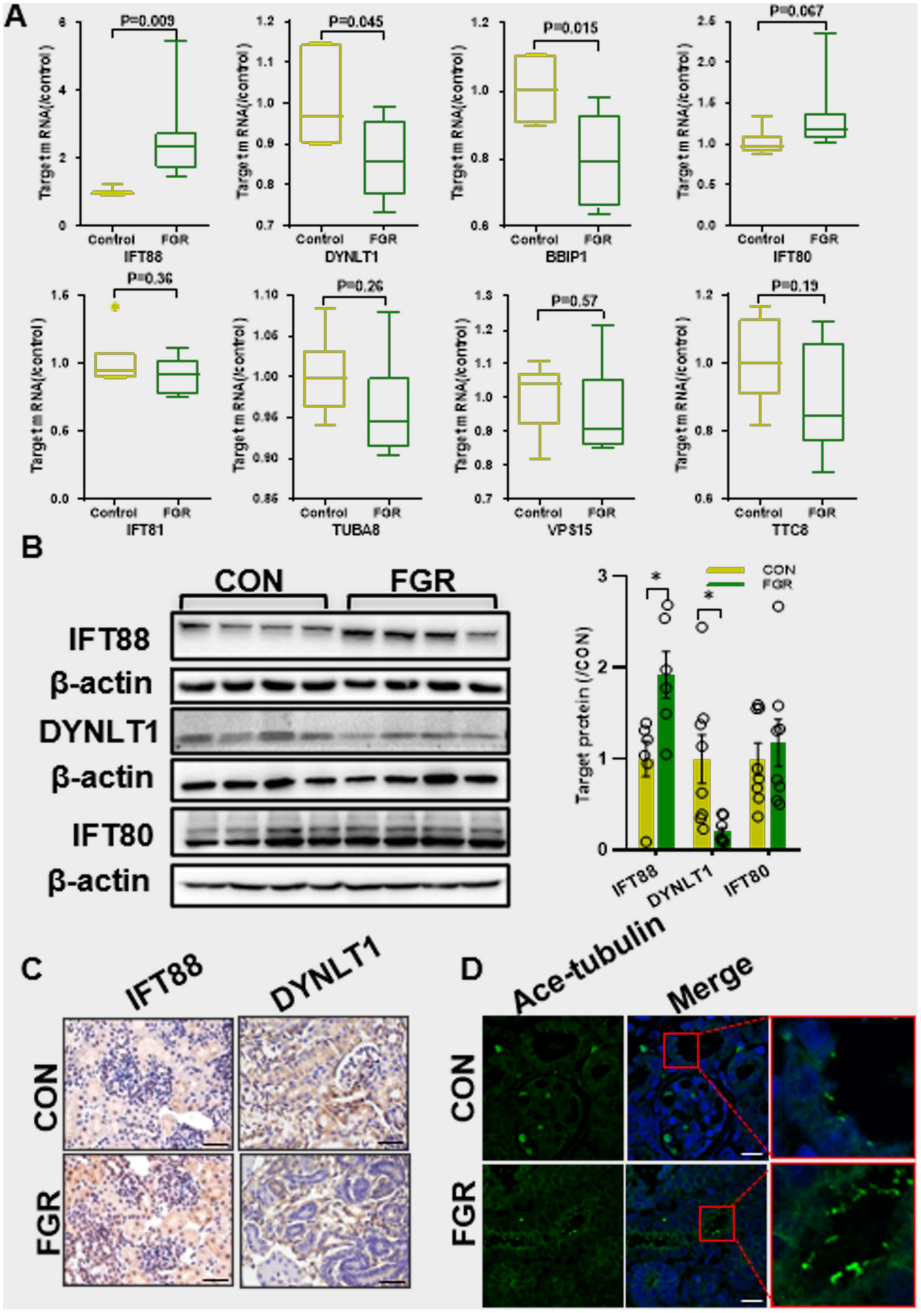
Effect of maternal protein restriction on primary cilia. **(A)** Q-PCR assay of the mRNA levels of ciliogenesis regulators (*n* = 5, *p < 0.05 vs. control). **(B)** WB assay of the protein expression of ciliogenesis regulators (*n* = 6–8 per group, *p < 0.05 vs. control). **(C)** IHC staining of IFT88 and DYNLT1 in fetal kidney (400×, scale bar = 100 μm). **(D)** IF staining of Ac-TUBA showed the elongated cilia in fetal kidney of FGR group (400×, scale bar = 100 μm).

Q-PCR assay showed that MPR caused an increase in IFT88 mRNA levels, a core protein in the IFTB complex. Instead the mRNA levels of Dynein light chain 1 (DYNLT1) and BBSome-interacting protein (BBIP) 1 was decreased in FGR fetal kidney. IFT80, another key component of IFTB complex, showed a slight but not significant increase in mRNA levels (Figure 2A). Western blot and IHC staining confirmed the alteration of IFT88 and DYNLT1 in protein levels. IHC staining further revealed that both DYNLT1 and IFT88 were mainly localized to tubular and less in glomerular epithelial cells (Figures 2B,C). IF staining of acetylated-tubulin (Ac-TUBA), a primary cilia marker, showed elongation of primary cilia in the renal tubular of FGR fetus (Figure 2D).

### 3.3. Effects of MPR on β-catenin pathway in fetal kidney

**Figure 3:**
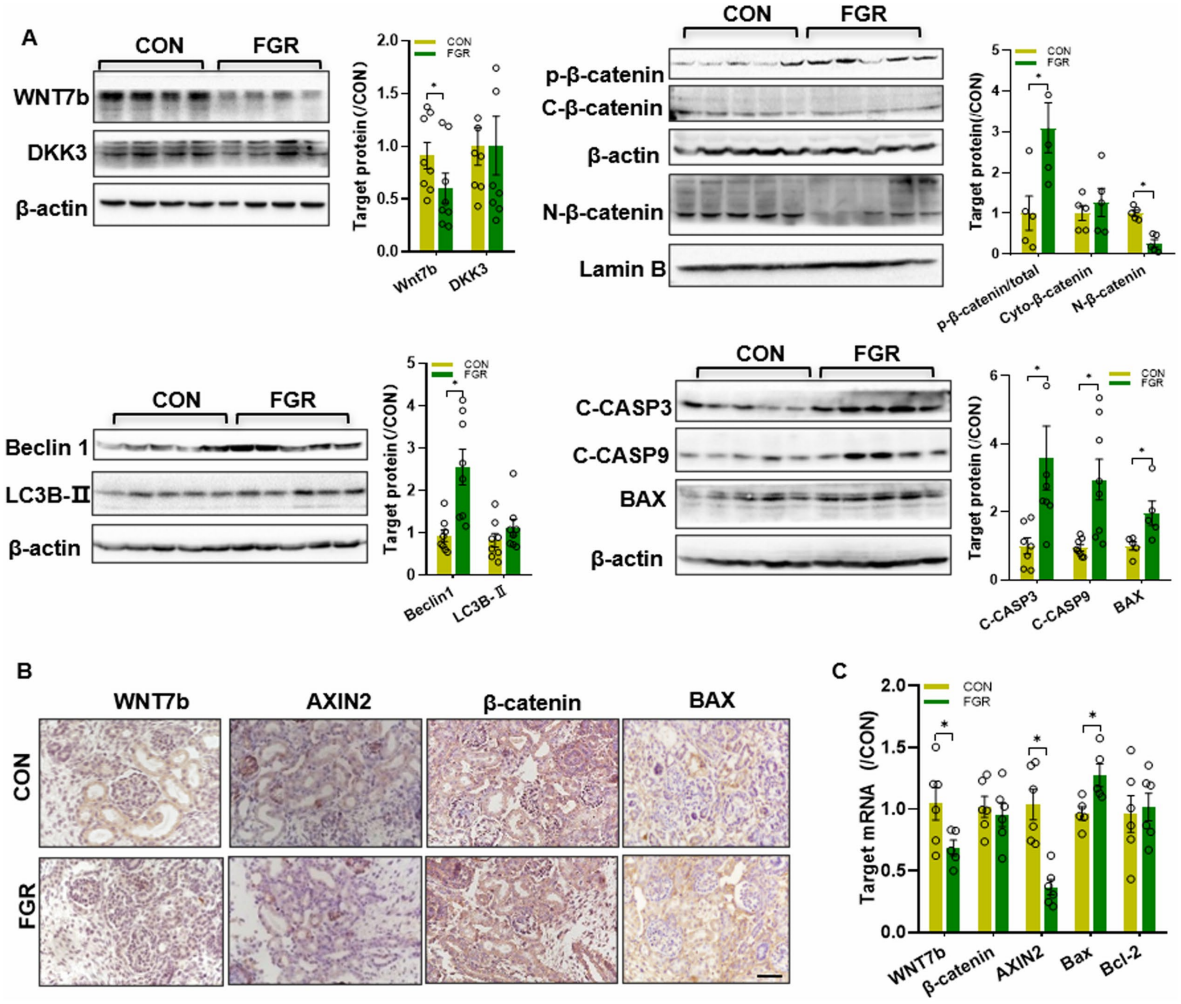
Effects of FGR onβ-catenin pathway. **(A)** WB assay of the β-catenin pathway, autophagy markers and apoptosis regulators in fetal kidney (*n* = 5–8, *p < 0.05 vs. control). **(B)** IHC staining of β-catenin pathway factors and Bax in the fetal kidney (400×, scale bar = 100 μm). **(C)** Q-PCR assay of the mRNA levels of Wnt pathway factors and apoptosis regulators in the fetal kidney (*n* = 6, *p < 0.05 vs. control).

WB assay showed a decrease in the Wnt7b protein level in FGR kidney (Figure 3A). The basal protein level of glycogen synthase kinase 3 β (GSK3β) was unchanged (data not shown). The basal protein level of β-catenin remained unaltered in cytoplasm, whereas the phosphorylated β-catenin (Ser33/37/T41) in cytoplasm was significantly elevated in FGR group. Instead, β-catenin nucleus translocation was remarkably reduced, suggestive of inhibition of β-catenin signaling. Dickkopf-related protein 3 (DKK3), a negative regulator of the Wnt pathway, was unchanged in FGR fetuses (Figure 3A). IHC staining showed that Wnt7b and Axin2(a β-catenin downstream target gene) were decreased in the kidney of FGR fetus (Figure 3B). The mRNA levels of Wnt7b and Axin2 were significantly downregulated, while β-catenin were unchanged, consistent with the protein data (Figure 3C).

### 3.4. Effects of MPR on renal autophagy and apoptosis regulators in fetal kidney

Beclin1 and LC3B are key regulators for autophagy. WB assay showed that renal Beclin1(an autophagy initiator) was upregulated in FGR fetus, yet LC3B (an autophagy marker) was unchanged. In addition, immunoblotting showed that pro-apoptotic factor Bax was upregulated in FGR fetuses. The anti-apoptotic factor Bcl-2 protein level was unchanged (data not shown). MPR also led to increased activation of apoptosis initiator caspase 9 and apoptosis executioner caspases 3 in the kidney of FGR fetus (Figure 3A). IHC staining also indicated that Bax protein was upregulated in FGR fetuses (Figure 3B). Changes in the mRNA levels of Bax and Bcl-2 were consistent with the protein data (Figure 3C).

### 3.5. Effects of overexpression of IFT88 on ciliogenesis and β-catenin signaling

**Figure 4:**
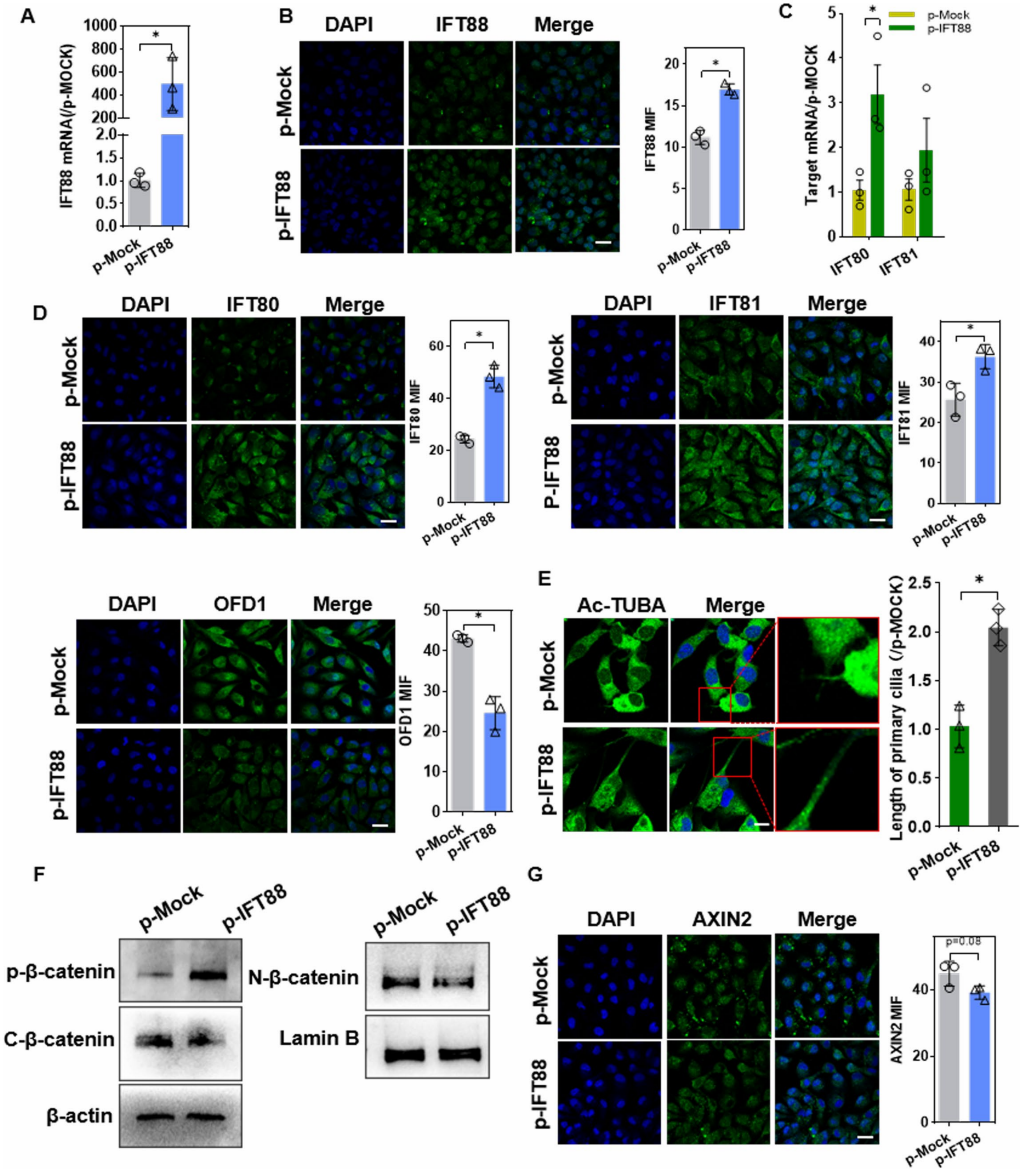
Effects of IFT88 overexpression on cilia ciliogenesis and β-catenin pathway. **(A,B)** Q-PCR assay **(A)**, and IF staining **(B)** to determine the expression efficiency in cells after transfection with p-IFT88 or MOCK plasmid for 48 h. *n* = 3; *p < 0.05. Blank: blank control; *p*-Mock: transfected with the empty vector; p-IFT88: transfected with pGV362-IFT88. (400×, scale bar = 100 μm). **(C)** Q-PCR assay of the ciliogenesis regulators IFT80 and 81 (*n* = 3, *p < 0.05 vs. p-Mock) **(D)** Representative photomicrographs and data of IF analysis of IFT80, 81 and OFD1 in cells (400×, scale bar = 100 μm) (*n* = 3, *p < 0.05 vs. p-Mock). **(E)** Ac-TUBA staining and measurement of the cilia length (×800 magnification, scale bar 200 μm) (*n* = 3, *p < 0.05 vs. p-Mock). **(F)** WB assay of β-catenin pathway factors. ‘C’ stands for cytosol and ‘N’ for nuclear, similarly hereinafter (*n* = 3, *p < 0.05 vs. p-Mock). **(G)** IF staining assay of Axin2 in HK-2 cells (400×, scale bar = 100 μm, *n* = 3, *p < 0.05 vs. p-Mock).

To further investigate this process, HK2 cells with transient IFT88 overexpression were generated by transfection of p-IFT88 plasmid, and gene overexpression efficiency was confirmed by q-PCR and IF staining (Figures 4A,B). IFT88 overexpression led to upregulation of IFT80/81, two critical components of IFTB complex, and caused downregulation of OFD1 (oral-facial-digital syndrome 1), a ciliogenesis suppressor (Figures 4C,D). Moreover, the cilia were elongated in p-IFT88-transfected cells (Figure 4E).

We further explored the effect of IFT88 on β-catenin activity. The basal level of β-catenin was decreased in cytoplasm, whereas the phosphorylation level was significantly elevated. Instead, β-catenin nucleus translocation was remarkably reduced (Figure 4F). IF staining showed a slight but not significant (*p* = 0.08) decrease in Axin2, the downstream target gene of β-catenin, in p-IFT88 group (Figure 4G).

### 3.6. Effects of overexpression of IFT88 on cell viability and cell autophagy and apoptosis

**Figure 5:**
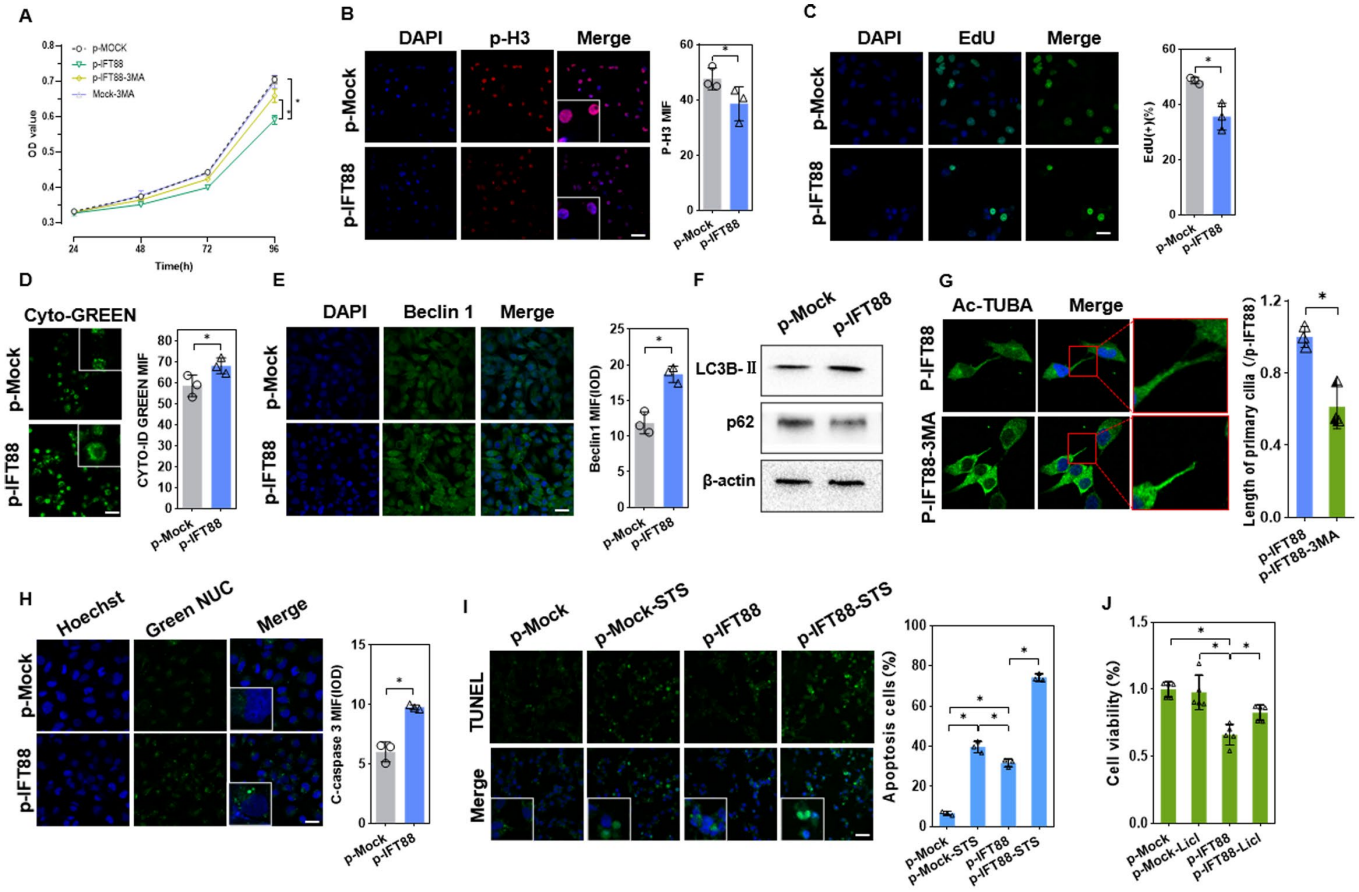
Effects of IFT88 overexpression on cell viability and cell death. **(A)** CCK8 assay of the cell viability of HK2 cells with or without 3MA (*n* = 5, *p < 0.05 vs. p-Mock). **(B)** IF staining of the proliferation marker p-H3 in HK2 cells (400×, scale bar = 100 μm, *n* = 3, *p < 0.05 vs. p-Mock). **(C)** EdU staining and assay of EdU-positive cells ratio (400×, scale bar = 100 μm, *n* = 3, *p < 0.05 vs. p-Mock). **(D)** Detection of autophagy vacuoles using Cyto-ID Green dye (400×, scale bar = 100 μm, *n* = 3, *p < 0.05 vs. p-Mock). **(E)** IF staining showed the expression of Beclin1 (400×, scale bar = 100 μm, *n* = 3, *p < 0.05 vs. p-Mock). **(F)** WB analysis of the autophagy regulators in cells (*n* = 3, *p < 0.05 vs. p-Mock). **(G)** Ac-TUBA staining and measurement of the cilia length after treatment with 3MA (800×, scale bar = 200 μm, *n* = 3, *p < 0.05 vs. p-Mock). **(H)** The caspase3/7 activity was measured by GreenNuc™ activity assay (400×, scale bar = 100 μm, *n* = 3, *p < 0.05 vs. p-Mock). **(I)** Apoptosis assay using TUNEL staining in HK-2 cells with or without apoptosis activator staurosporine (STS) (400×, scale bar = 100 μm, *n* = 3, *p < 0.05 vs. p-Mock). **(J)** CCK8 assay of the cell viability in HK2 cells with or without Licl, a β-catenin pathway activator (*n* = 5, *p < 0.05 vs. p-Mock).

CCK8 assay showed that IFT88 overexpression reduced the cell viability (Figure 5A). IF staining showed that phosphorylated Histone H3 (p-H3), a mitosis marker, was downregulated in p-IFT88 transfection cells. To further observe cell proliferation, we used the EdU Cell Proliferation Assay Kit. EdU incorporation assay also confirmed that overexpression of IFT88 suppressed the cell proliferation (Figures 5B,C).

We further analyzed whether IFT88 influences autophagy activation. IFT88 overexpression led to enhanced Cyto-ID® Green dye fluorescence signals, suggesting an increase of autophagosomes (Figure 5D). IF staining showed that Beclin1, an autophagy initiator, was upregulated in p-IFT88 transfection cells (Figure 5E). As expected, IFT88 overexpression caused an increase in LC3B-II and a decrease in p62 protein (Figure 5F). Further analysis revealed that 3-MA, a well-accepted autophagy inhibitor, partially restored the cell viability in IFT88 overexpression cells (Figure 5A), which indicated that IFT88 overexpression caused autophagy-dependent cell death. Meanwhile, IF staining showed that 3-MA restored cilium length to normal level (Figure 5G), suggesting that the effect of IFT88 on ciliogenesis was autophagy-dependent.

A further analysis was performed to investigate the effect of IFT88 on cell apoptosis. Activation of caspase 3/7 was detected by fluorescence microscopy with Green NUC Reagent. The activity of caspase 3/7 was enhanced at 48 h post transfection (Figure 5H). We further determined the cell apoptosis induced by staurosporine (STS), an apoptosis activator. TUNEL staining analysis showed that IFT88 overexpression alone moderately induce apoptosis, while it potently aggravated STS-induced apoptosis in HK-2 cells (Figure 5I). Meantime, CCK8 assay revealed that treatment with LiCl, a β-catenin pathway activator, partially restored the cell viability in IFT88 overexpressed cells (Figure 5J), which indicated that inhibition of β-catenin signaling is at least partly responsible for cell apoptosis induced by IFT88 overexpression. Taken together, the above results demonstrated that IFT88 overexpression inhibited β-catenin signaling, promoted autophagy, and decreased the anti-apoptosis potential.

### 3.7. Effects of DYNLT1 knockdown on primary cilia and β-catenin signaling

**Figure 6:**
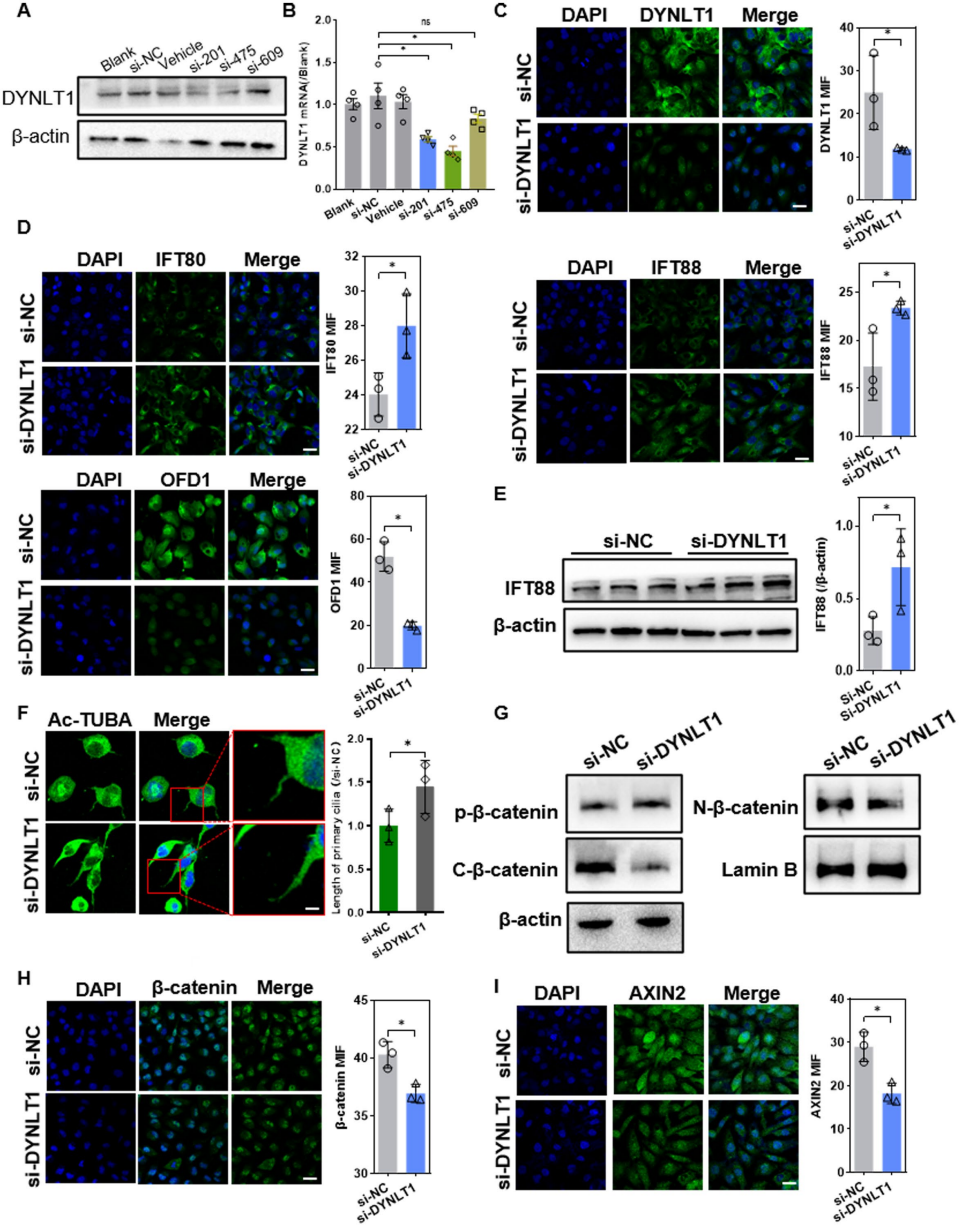
Effects of DYNLT1 knockdown on ciliogenesis and Wnt pathway. **(A–C)** Western blot **(A)**, Q-PCR assay **(B)** and IF staining **(C)** (400×, scale bar = 100 μm,) to determine the knockdown efficiency of si-DYNLT1 (*n* = 3, *p < 0.05 vs. si-NC). **(D)** IF staining showed the expression of IFT88, 80 and OFD1 (400×, scale bar = 100 μm, *n* = 3, *p < 0.05 vs. si-NC). **(E)** WB assay showed the upregulation of IFT88 after transfection with si-DYNLT1(*n* = 3, *p < 0.05 vs. si-NC). **(F)** IF staining of Ac-TUBA and measurement of the cilia length (800×, scale bar = 200 μm, *n* = 3, *p < 0.05 vs. si-NC). **(G)** WB assay of the expression of β-catenin pathway factors in HK2 cells (*n* = 3, *p < 0.05 vs. si-NC). **(H-I)** IF staining assay of β-catenin and Axin2 in HK2 cells (400×, scale bar = 100 μm, *n* = 3, *p < 0.05 vs. si-NC).

To further investigate the effect of DYNLT1 on cilia, the DYNLT1 gene was silenced with siRNA in HK2 cell. Knockdown (KD) efficiency of si-DYNLT1 was confirmed with qPCR, WB and IF (Figures 6A–C). As si-201 and si-475 showed similar efficiency in gene expression silencing, the two siRNA were pooled together in equal proportions to form a mixture. WB assay and IF staining revealed that downregulation of DYNLT1 increased the protein abundance of IFT88 and 80, decreased the protein level of OFD1(Figures 6D,E). IF staining of Ac-TUBA showed that DYNLT1 KD led to lengthening cilia (Figure 6F). We further explored the effect of DYNLT1 on β-catenin activity. The basal level of β-catenin was decreased in cytoplasm, in contrast the phosphorylation level was significantly elevated in DYNLT1 KD cells. Meanwhile, β-catenin nucleus translocation was remarkably reduced, evidenced by WB and IF staining. Axin2, a target gene of β-catenin, was downregulated in DYNLT1 KD cells (Figures 6G–I).

### 3.8. Effects of DYNLT1 knockdown on cell viability

**Figure 7:**
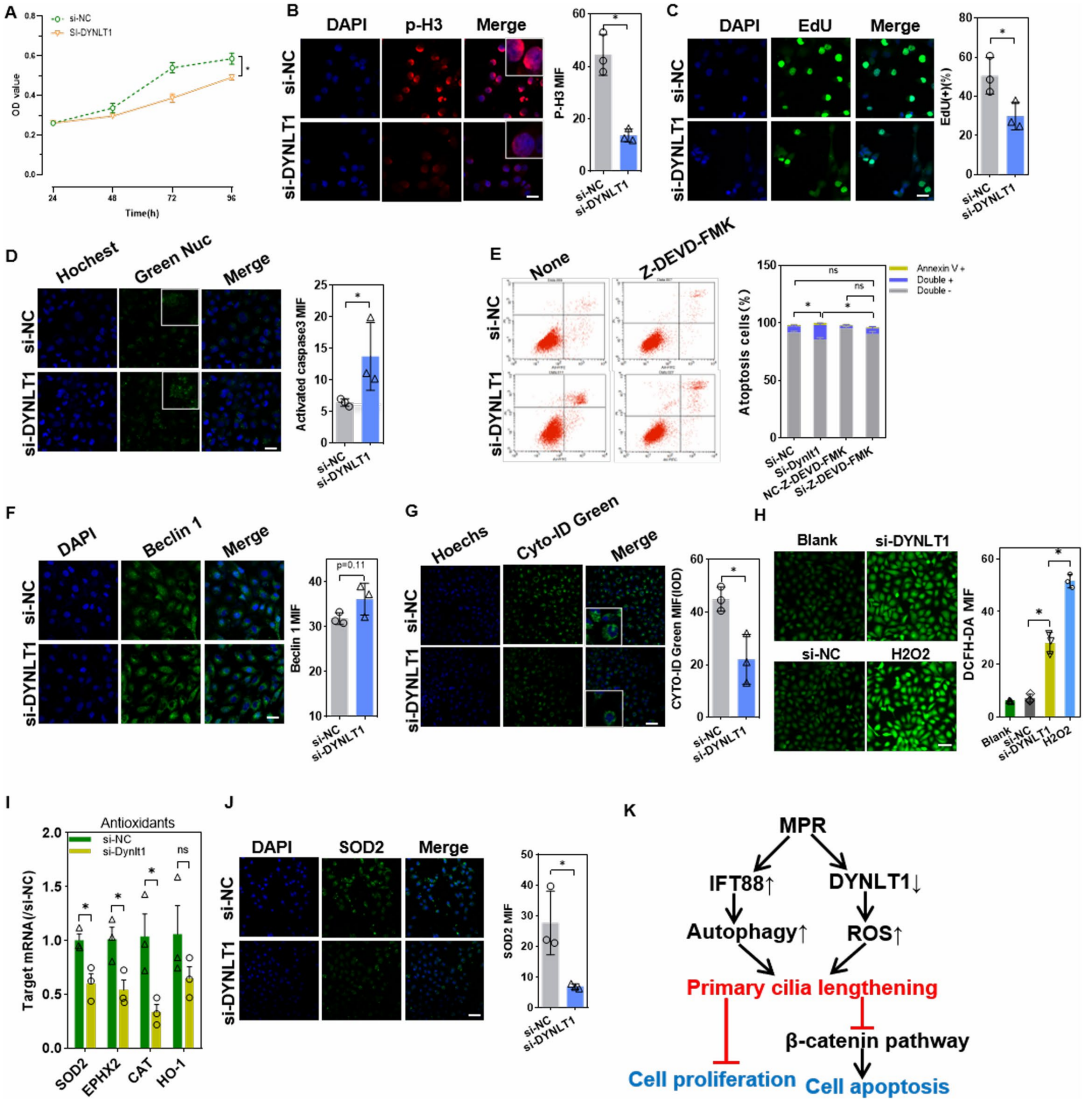
Effects of DYNLT1 suppression on cell viability and apoptosis. **(A)** CCK8 assay of the cell viability after siRNAs transfection (*n* = 5, *p < 0.05 vs. si-NC). **(B)** IF staining of the proliferation marker p-H3 in HK2 cells (400×, scale bar = 100 μm, *n* = 3, *p < 0.05 vs. si-NC). **(C)** EdU staining and assay of EdU-positive cells ratio (400×, scale bar = 100 μm, *n* = 3, *p < 0.05 vs. si-NC). **(D)** The caspase3/7 activity were detected with GreenNuc™ staining (400×, scale bar = 100 μm, *n* = 3, *p < 0.05 vs. si-NC). **(E)** Cell apoptosis assay using an Annexin V-PI kit in HK-2 cells with or without caspase 3 inhibitor (*n* = 3, ∗*p* < 0.05, vs. si-NC with or without Z-DEVD-FMK). **(F)** IF staining showed the expression of the autophagy initiator Beclin1(400×, scale bar = 100 μm, *n* = 3, *p < 0.05 vs. si-NC). **(G)** CYTO-ID Green dye staining showed the autophagy vesicles (400×, scale bar = 100 μm, *n* = 3, *p < 0.05 vs. si-NC). **(H)** DCFH-DA probe assay showed the cytoplasmic ROS level. H_2_O_2_(20 μM) treatment was used as a positive control (200×, scale bar = 50 μm, *n* = 3, *p < 0.05 vs. si-NC). **(I)** Q-PCR assay of the mRNA levels of intracellular antioxidant factors (*n* = 3, *p < 0.05 vs. si-NC). **(J)** IF staining showed the expression of the antioxidant enzyme SOD2 (200×, scale bar = 50 μm, *n* = 3, *p < 0.05 vs. si-NC). **(K)** A schematic representation of study findings.

CCK8 assay showed that DYNLT1 KD decreased the cell viability (Figure 7A). Cell mitosis marker p-H3 was reduced in KD cells, suggesting a decrease in cell proliferation. EdU assay revealed a decrease in EdU+ cells after silencing of DYNLT1 (Figures 7B,C). Furthermore, confocal analysis and FCM assay revealed that DYNLT1 deficiency increased the caspase 3/7 activity, and led to an increase in late apoptosis and necrotic cells (Annexin V+/PI+). The caspase-3 inhibitor z-DEVD-FMK markedly inhibited DYNLT1 KD-induced cell death (Figures 7D,E). In contrast to IFT88 overexpression, DYNLT1 deficiency had diverse effects on autophagy reaction. The autophagy initiation factor Beclin1 showed a slight but not significant increase in DYNLT1 KD cells (Figure 7F). In contrast, Cyto-ID® Green staining showed a decrease in autophagosomes in KD group (Figure 7G). The cilia elongation induced by DYNLT1 KD seems independent of autophagy. To further explore the mechanism underlying elongated cilia, we further determined the cytoplasmic ROS level with DCFH-DA probe, for ROS have been reported to play a stimulatory role in the elongation of cilia (19). Interestingly, DCFH-DA staining showed a higher accumulation of ROS in DYNLT1 KD cells (Figure 7H). Simultaneously, DYNLT1 KD downregulated the expression of antioxidants catalase (CAT), superoxide dismutase (SOD)2 and epoxide hydrolase (EPHX)2, which may serve as an explanation for increased ROS level (Figures 7I,J). Figure 7K shows a schematic representation of working hypothesis.

## 4. Discussion

Deficient intrauterine environment, such as ischemia, hypoxia, and nutrient deficiencies, impairs developing organ architecture and its biological functional, subsequently leads to increased susceptibility to disease in adults (3, 20). Programmed hypertension of fetal origin is associated with renal hypoplasia, yet, the underlying pathogenesis remains unknown (21). The main finding of present study includes: (i) MPR impacted both glomeruli and tubuli development, consistent with previous reports (22, 23). (ii) IFT88, a positive regulation factor for ciliogenesis was increased, in contrast, negative regulation factor DYNLT1 was decreased in FGR fetal kidney. Meanwhile, β-catenin signaling pathway was inhibited and apoptosis was activated in fetal kidney. (iii) Overexpression of IFT88 resulted in elongated cilia and caused inhibition of the β-catenin signaling in HK2 cells, subsequently led to cell apoptosis and autosis. (iv) DYNLT1 suppression increased cytosolic ROS levels, caused longer cilia, and led to cell apoptosis, without autophagy activation.

Primary cilia are present in various tissues throughout vertebrate development and act as cellular antennae to mediate cell signaling. Numerous ciliopathies are associated with defects in cilia length caused by deregulation of ciliary associated proteins (24, 25). Accumulating evidence has confirmed the presence of primary cilia in developing and mature kidneys. The role of primary cilia in kidney injury has been confirmed. The cilia initially shorten in the proximal tubules and then lengthen in the proximal and distal tubules after ischemia–reperfusion injury. Ureteral obstruction leads to elongated cilia in the distal tubule (26). The cilia also play vital roles in nephrogenesis and presumably establishment of fetal kidney functions later during embryogenesis (27, 28). The cilia length is proposed to modulate cilia function. There is a balance between cilia length and intraflagellar transport (IFT). The IFT particles and their associated cargo proteins are transported along axonemal microtubules by kinesin-2 motor proteins in the anterograde direction and by cytoplasmic dynein-2 in the retrograde direction. As a critical component of IFT-B particle, IFT88 is essential for lengthen microtubules axoneme, as well as anterograde trafficking within the cilium. IFT88 knockdown leads to dotted and shorter primary cilia in osteoblasts (29). IFT88 knockout severely impairs renal autophagy in mice (30). Conversely, DYNLT1 functions as a negative regulator of ciliogenesis. Knockout of DYNLT1 blocks dynein-2 function, and then causes elongated cilia in human retinal pigment epithelium cell line RPE1 (31). Consistent with our hypothesis, the present study identified upregulation of IFT88 and downregulation of DYNLT1 accompanied with aberrant longer cilia in FGR fetal kidney. *In vitro* study showed that IFT88 overexpression elongated the cilia, suppressed cell proliferation, and induced cell apoptosis. Previous reports show that elimination of primary cilia by chemical compound or by gene silencing promotes cells proliferation (32, 33). Consistently, we showed here that aberrant longer cilia suppressed cell proliferation. Additionally, IFT88 overexpression enhanced autophagy flux, and decreased the protein amount of OFD1. OFD1 acts as a suppressor of ciliogenesis, while removal of OFD1 from centriolar satellites by autophagy degradation promotes ciliogenesis (34). Excessive accumulation of OFD1 due to dysregulation of autophagy causes fewer ciliated cells in the endometrial cancer tissues (35). Like the changes of overexpressed cells, the autophagy initiator Beclin1 was upregulated in FGR fetus, while LC3B showed no significant changes, which may be due to the composition of different types of cells in renal tissue. The activation of renal autophagy in fetal rat cannot be determined. Our results support the hypothesis that aberrant upregulation of IFT88 increases IFTB complex, promote OFD1 autophagic degradation, and elongates primary cilia in the tubular of FGR fetus. DYNLT1 knockdown also caused longer cilia and inhibited cell proliferation. However, DYNLT1 KD lengthen the cilia without activating autophagy. Instead, it downregulated antioxidant factors and led to an increase in ROS. H_2_O_2_ treatment elongates primary cilia in kidney tubular epithelial cells (19). We therefore speculate that cilia lengthening in DYNLT1 cells is due to ROS accumulation, which warrants further research.

Primary cilia modulate tubular epithelial cell maturation during embryonic stage by regulating Wnt signaling pathways (36, 37), for various Wnt pathway proteins, including degradation complexes and calmodulin regulator planar cell polarity (PCP) protein, are localized to the cilia or the basal body. IFT88 knockdown leads to a decrease in β-catenin phosphorylation and promotes nuclear translocation of β-catenin (15). Dysfunction of cilia caused by PKD2 knockout in mice leads to overexpression of Wnt7a/7b and activation of the Wnt/β-catenin pathway in the kidneys (10). Our results demonstrated that IFT88 overexpression or DYNLT1 knockdown increased the phosphorylation of β-catenin, simultaneously decreased the accumulation of β-catenin in nucleus. We suggested that deregulation in ciliogenesis factors led to aberrant enlonged primary cilia, then inhibited β-catenin signaling.

The canonical β-catenin pathway molecules present in the three critical stages of embryonic kidney development (pro-, meso-, and metanephrons), and are instrumental for UB induction, nephron formation, and maturation (9, 38). Wnt7b, a key ligand for canonical Wnt signaling,is closely related to the formation and differentiation of renal vascular endothelium (39, 40). Wnt7b locates in the UB during the early stages of development, and promotes the formation of capillaries around the UB. Deletion of Wnt7b cause the embryonic kidneys fail to form the medullary zone and are unable to concentrate urine normally (41). Wnt7b^−/−^ downregulates the target gene Axin2 in the medullary stromal cells near the collecting duct epithelium, by inhibiting β-catenin activity (42, 43). Activation of Wnt/β-catenin signaling can inhibit or promote apoptosis, depending on the cell type (44). In the kidneys, Wnt/β-catenin signaling regulates Bax-mediated apoptosis after induction of metabolic stress. Constitutively active β-catenin decreases Bax activation in the tubular epithelial cells and significantly reduces cell apoptosis after metabolic stress (45). We speculated that increased degradation of β-catenin upregulated pro-apoptosis factor Bax, induce caspase-dependent apoptosis in FGR fetal rat kidneys. This may at least partly responsible for the tubular dysplasia and impaired renal function in adult rats.

### Limits of this study

The present study only investigated the changes in the kidney of near-term fetus, without revealing the changes during growth and branching of the ureteric bud period. It thus cannot be ruled out the role of primary cilia in the reduction of glomerular numbers. Further, cilia proteins (e.g., IFT88) localize in other sites and non-ciliated cells, and have non-ciliary functions, including cytoskeletal regulation and trafficking (46). A non-ciliated effect of IFT88 overexpression on kidney development warrants continued study. In addition, how DYNTL deficiency-induced ROS accumulation impact the ciliogenesis remain to be further investigated.

In conclusion, our results revealed that intrauterine protein malnutrition led to deregulation of ciliogenesis factors and longer cilia in renal tubular epithelial. Abnormal elongated cilia inhibited canonical Wnt/β-catenin signaling, and induced cell apoptosis in fetal kidney tubular. This could be at least in part responsible for the maldevelopment of kidney and increased risks for hypertension in adulthood.

## Data availability statement

The original contributions presented in the study are included in the article/Supplementary material, further inquiries can be directed to the corresponding author.

## Ethics statement

The animal study was reviewed and approved by Animal Research Committee of China Medical University.

## Author contributions

JW, PZ, LZ, and HG performed the experiments and statistical analysis. XL and JG contributed to conception and design of the study and wrote the manuscript. All authors contributed to the article and approved the submitted version.

## Funding

This work was supported by grant from the National Natural Science Foundation of China (no. 81971400), Outstanding Scientific Fund of Shengjing Hospital (no. 201707), Natural Fund Project of Liaoning Province (no. 2020-MS-158), and People’s Livelihood Science and Technology Project of Liaoning Province (2021JH2/10300126).

## Conflict of interest

The authors declare that the research was conducted in the absence of any commercial or financial relationships that could be construed as a potential conflict of interest.

## Publisher’s note

All claims expressed in this article are solely those of the authors and do not necessarily represent those of their affiliated organizations, or those of the publisher, the editors and the reviewers. Any product that may be evaluated in this article, or claim that may be made by its manufacturer, is not guaranteed or endorsed by the publisher.

## Abbreviations

MPR: maternal protein restriction
FGR: fetal growth restriction
IFT: intraflagellar transport
DYNLT1: dynein light chain tctex-type 1
PAS: periodic acid–schiff
IHC: immunohistochemistry
IF: immunofluorescence
UB: ureteric bud
DKK3: dickkopf-related protein 3
AQP: aquaporin
GSK3β: glycogen synthase kinase 3 β
OFD1: oral-facial-digital syndrome 1
PKD: polycystic kidney disease
DAPI: 4′,6-diamidino-2-phenylindole
siRNA: small interfering RNA
Ac-TUBA: acetylated tubulin alpha
STS: staurosporine
3-MA: 3-methyladenine
MAP1LC3/LC3: microtubule-associated protein 1 light chain 3
AXIN2: Axis inhibition protein2
ROS: reactive oxygen species
CAT: catalase
SOD: superoxide dismutase
EPHX: epoxide hydrolase
