# Supplementary material for: Maternal protein deficiency alters primary cilia length in renal tubular and impairs kidney development in fetal rat
Source: Front Nutr. 2023 Jul 6;10:1156029. doi: 10.3389/fnut.2023.1156029 (PMC10358357; doi:10.3389/fnut.2023.1156029)
Supplement: Supplementary file 1 [file Data_Sheet_1.docx]

Supplementary Material

Maternal protein deficiency alters primary cilia length in renal tubular and impairs kidney development in fetal rat

**Jun Wang^1^†, Pei Zhou^1^†, Liangliang Zhu^1^†, Hongbo Guan^1^, Jian Gou^2^*, Xiaomei Liu^1^** *Correspondence:**

Xiaomei Liu, E-mail: [liuxm@cmu.edu.cn](mailto:liuxm@cmu.edu.cn)

# Supplementary Tables

**1.1 Supplementary Table 1. Ingredients of rat diets.**

|  | **Control diet (%)** | **LP diet (%)** |
| --- | --- | --- |
| Protein | 22.2 | 7 |
| Carbohydrates | 61.2 | 74.7 |
| Fat | 4.8 | 6.9 |
| Ca | 1.62 | 0.53 |
| P | 0.92 | 0.23 |
| Calories | 399 kcal/100 g | 395 kcal/100 g |

LP, low protein

**1.2** **Supplementary Table 2. Sequences of primers and siRNA**

| **Gene name** | | **Accession number** | **Primer sequences (5’-3’)** |
| --- | --- | --- | --- |
| **Rat primary cilia** | | |  |
| **IFT80** | NM_001013911 | | AACATCCAGGAGGCTGAAAC  CAGTGCCCTTTCCCAGTTAT |
| **IFT88** | NM_001107266 | | TTCAGCAAGCAGTGAGAACC  AGGGATGTCTTGGACCCATA |
| **BBIP1** | 6498801 | | AACAAGGGCAGTTGTCTGTG  TCTTGTTGGCGAACTGTGTC |
| **TUBA8** | NM_001024339 | | GGTGATGTGGTACCCAAGGA  TAATGCCCACCTTGAAGCCT |
| **DYNLT1** | [NM_031318.1](https://www.ncbi.nlm.nih.gov/nuccore/NM_031318.1) | | TCCGAGGAGACTGCATTTGT  CTGGTTGACTTTGCTGTGCT |
| **TTC8** | [XM_006240423.3](https://www.ncbi.nlm.nih.gov/nuccore/XM_006240423.3) | | CTCAAGCTGTCAGGCCAATC  TTGTACCCGGTCTTCCACTC |
| **TULP4** | [NM_001109137.1](https://www.ncbi.nlm.nih.gov/nuccore/NM_001109137.1) | | CACGTGTGATGCAGATGGAG  GAACCAACCAGGACAAACCC |
| **VPS15** | NM_001401214.1 | | CGTGTTGGGACATGAGGTTC  GCCCTGAACAGCTGCAATTA |
| **Rat classic β-catenin pathway** | | |  |
| **WNT7a** | | NM_001100473.1 | GCGCTCTAGGACAGTCTCCA  GGGGCAATCCACATAGCCTG |
| **WNT7b** | | NM_001009695.1 | AGCCAACATCATCTGCAACA  GGCATTCATCGATACCCATC |
| **DKK3** | | NM_138519.2 | CACACAGCCACAGTCTGGTA  CTCCTCAAATGCCATCTCCT |
| **GSK3β** | | NM_019827.6 | AGTGCCACTCAGCAACACTG  CCCCACCTCTTGGATCTCCT |
| **β-catenin** | | NM_001165902.1 | ACAGCACCTTCAGCACTCT-3  AAGTTCTTGGCTATTACGACA-3 |
| **AXIN2** | | NM_024335.1 | CTGGCTATGTCTTTGCACCA -3  AGGAGGGATTCCATCTACGC-3 |
| **Rat apoptosis regulators** | | |  |
| **Bax** | | NM_007527.3 | AAACTGGTGCTCAAGGCCCT  5-AGCAGCCGCTCACGGAG |
| **Bcl-2** | | NM_016933.1 | CCGGGAGAACAGGGTATGATAA  CCCACTCGTAGCCCCTCTG |
| **β-actin** | | NM_031144.3 | AGTCCCTCACCCTCCCAAAAG  AAGCAATGCTGTCACCTTCCC |
| **Human primers** | | |  |
| **IFT88** | | NM_175605.5 | GTTATGATTGGTGCGTGGAAGT  GGGCTGAGAGATTGGTTGCAG |
| **IFT80** | | NM_020800.3 | GTGGGGCCCTGATTCAGAAA  GCGGCCGTAACTATCCCATA |
| **IFT81** | | NM_025132.4 | TGAAGATGGATGACCGAACCT  GCAGACAATGTTGCCAAAGTC |
| **DYNLT1** | | NM_006519.4 | GTTCTCCACTCAATACAGGGTG  TGGCTGGTGGTTAGAGGATG |
| **OFD1** | | NM_003611.3 | ACCAGACGTTTAAGGATCGGG  GTTCTCCACTCAATACAGGGTG |
| **CAT** | | NM_001752 | TGGGATCTCGTTGGAAATAACAC  TCAGGACGTAGGCTCCAGAAG |
| **EPHX2** | | [NM_022936.1](https://www.ncbi.nlm.nih.gov/nuccore/NM_022936.1) | GAGGTACCAGATCCCTGCTC  ACCATCTCCTCACACAGCAA |
| **HO1** | | [NM_012580.2](https://www.ncbi.nlm.nih.gov/nuccore/NM_012580.2) | GCATGTCCCAGGATTTGTCC  ACCAGCTTAAAGCCTTCCCT |
| **SOD2** | | [NM_017051.2](https://www.ncbi.nlm.nih.gov/nuccore/NM_017051.2) | CCGAGGAGAAGTACCACGAG  TAGGGCTCAGGTTTGTCCAG |
| **SiRNA sequence** | | |  |
| **Si-Dynlt1** | | Si-201 | GGACCACAAAUGUAGUAGATT  UCUACUACAUUUGUGGUCCTT |
|  | | Si-475 | CAGCCAUGAAUUCAGUGAATT  UUCACUGAAUUCAUGGCUGTT |
|  | | Si-619 | GUUGCCACUUGUCUUAACUTT  AGUUAAGACAAGUGGCAACTT |
| **Si-NC** | |  | UUCUCCGAACGUGUCACGUTT  ACGUGACACGUUCGGAGA ATT |
| **pGV417-IFT88 PCR identification primers** | | | ATTCCAACCGATCCTCAAGT  AACGCACACCGGCCTTATTC |

**1.3 Supplementary Table 3. Antibodies for western blotting and IHC.**

| **Antibodies** | Source | Catalog number |
| --- | --- | --- |
| IFT88 | Proteintech | 60227-1-lg |
| IFT80 | Proteintech | 25230-1-AP |
| IFT81 | Proteintech | 11744-1-AP |
| OFD1 | Proteintech | 22851-1-AP |
| DYNLT1 | Proteintech | 11954-1-AP |
| Wnt7a | Proteintech | 10605-1-AP |
| Wnt7b | ThermoFisher | PA5-41979 |
| Dkk3 | Proteintech | 10365-1-AP |
| p-β-catenin | Wanleibio | WL03554 |
| β-catenin | Enogene | E1A6266 |
| AXIN2 | Proteintech | 20540-1-AP |
| PCNA | Proteintech | 10205-2-AP |
| AQP1 | Proteintech | 20333-1-AP |
| AC-TUBA | Proteintech | 66200-1-Ig |
| Bax | Proteintech | 60267-1-lg |
| Bcl-2 | Enogene | E1A6139 |
| Caspase3 | Absin | Abs132005 |
| Caspase9 | Enogene | E1A5244 |
| Cyclin D1 | Abclonal | A19038 |
| Cyclin E | Abclonal | A12000 |
| LC3B | CST | 2775 |
| Beclin1 | Proteintech | 11306-1-AP |
| P62 | Abclonal | A7758 |
| Laminb | Wanleibio | WL01775 |
| β-actin | Proteintech | 60008-1-lg |

**1.4 Supplementary Table 4. Kidney parameters of adult offspring.**

| Parameters | CON | FGR |
| --- | --- | --- |
| **Body weight(g)** | 335.21±28.19 | 450.63±33.99^*^ |
| **Renal weight(g)** | 2.23±0.25 | 2.33±0.42 |
| **N-acetyl-β-D-glucosaminidase (U/L)** | 3.47±1.80 | 17.49±8.81^*^ |
| **Urine protein (g/L)** | 0.31±0.08 | 0.90±0.36^*^ |
| **Blood urea (umol/L)** | 6.92±0.78 | 9.31±0.73^*^ |
| **Systolic pressure (mmHg)** | 130.43±10.39 | 151.63±19.41^*^ |

Compare with CON, *p<0.05.
